# Phylogenomic Analyses Reveal an Allopolyploid Origin of Core Didymocarpinae (Gesneriaceae) Followed by Rapid Radiation

**DOI:** 10.1093/sysbio/syad029

**Published:** 2023-05-09

**Authors:** Lihua Yang, A J Harris, Fang Wen, Zheng Li, Chao Feng, Hanghui Kong, Ming Kang

**Affiliations:** Key Laboratory of Plant Resources Conservation and Sustainable Utilization, South China Botanical Garden, Chinese Academy of Sciences, Guangzhou 510650, China; Key Laboratory of Plant Resources Conservation and Sustainable Utilization, South China Botanical Garden, Chinese Academy of Sciences, Guangzhou 510650, China; Guangxi Institute of Botany, Guangxi Zhang Autonomous Region and the Chinese Academy of Sciences, 541006 Guilin, China; Department of Ecology and Evolutionary Biology, University of Arizona, 1041 E. Lowell St., Tucson, AZ 85721, USA; Key Laboratory of Plant Resources Conservation and Sustainable Utilization, South China Botanical Garden, Chinese Academy of Sciences, Guangzhou 510650, China; Key Laboratory of Plant Resources Conservation and Sustainable Utilization, South China Botanical Garden, Chinese Academy of Sciences, Guangzhou 510650, China; Key Laboratory of Plant Resources Conservation and Sustainable Utilization, South China Botanical Garden, Chinese Academy of Sciences, Guangzhou 510650, China

**Keywords:** Hybridization, incomplete lineage sorting, lamiales, phylogeny, transcriptome, whole-genome duplication

## Abstract

Allopolyploid plants have long been regarded as possessing genetic advantages under certain circumstances due to the combined effects of their hybrid origins and duplicated genomes. However, the evolutionary consequences of allopolyploidy in lineage diversification remain to be fully understood. Here, we investigate the evolutionary consequences of allopolyploidy using 138 transcriptomic sequences of Gesneriaceae, including 124 newly sequenced, focusing particularly on the largest subtribe Didymocarpinae. We estimated the phylogeny of Gesneriaceae using concatenated and coalescent-based methods based on five different nuclear matrices and 27 plastid genes, focusing on relationships among major clades. To better understand the evolutionary affinities in this family, we applied a range of approaches to characterize the extent and cause of phylogenetic incongruence. We found that extensive conflicts between nuclear and chloroplast genomes and among nuclear genes were caused by both incomplete lineage sorting (ILS) and reticulation, and we found evidence of widespread ancient hybridization and introgression. Using the most highly supported phylogenomic framework, we revealed multiple bursts of gene duplication throughout the evolutionary history of Gesneriaceae. By incorporating molecular dating and analyses of diversification dynamics, our study shows that an ancient allopolyploidization event occurred around the Oligocene–Miocene boundary, which may have driven the rapid radiation of core Didymocarpinae.

Whole-genome duplication (WGD), or polyploidy, has occurred commonly in land plant evolution, with up to 35% of vascular plant species being nascent polyploids (Wood et al. 2009). Recent genomic analyses suggest that all angiosperms may have undergone at least two rounds of WGD in their history (Jiao et al. 2011; Li et al. 2015; One Thousand Plant Transcriptomes Initiative 2019). Whereas it is generally accepted that WGD is an important force in shaping the evolutionary history of angiosperms, the frequency and phylogenetic occurrence of WGD events in nature remain to be fully revealed, due to limitations in both methodology and sampling.

Rapid advances in transcriptome and genome sequencing and accompanying bioinformatics methods have greatly improved our ability to identify and place WGD events within the tree of life (Cannon et al. 2015; Edger et al. 2015; Li et al. 2015; Huang et al. 2016; Ren et al. 2018; Yang et al. 2018; Cai et al. 2019; One Thousand Plant Transcriptomes Initiative 2019; Wang et al. 2019; Zhang et al. 2020b). Until now, studies using these methods have mostly focused on deep phylogenetic relationships with consequently limited sampling. Ideally, investigation of the macroevolutionary consequences of WGDs requires relatively extensive sampling of genomes or transcriptomes within a specific plant clade. To date, only a few studies fulfill this requirement (Huang et al. 2016; Yang et al. 2018; Zhao et al. 2021).

Hybridization, whether accompanied by WGD or not, is widely regarded as a creative evolutionary force, increasing genomic diversity and facilitating adaptation and speciation (Seehausen 2004; Mallet 2005). WGDs may have either autopolyploid or allopolyploid origins (Soltis and Soltis 2009; Barker et al. 2016), and these two possible origins are often not disentangled in studies of the effects of WGDs on diversification (Mayrose et al. 2011; Tank et al. 2015; Landis et al. 2018; Ren et al. 2018; Smith et al. 2018; but see Estep et al. 2014). Differentiating autopolyploid or allopolyploid origins within the context of evolutionary history and modern biodiversity may yield insights into the roles of WGD in the adaptation and diversification of plants (Soltis et al. 2010; Madlung 2013).

Allopolyploids arise via hybridization between parental plants with different adaptive norms, usually different species, accompanied by genome doubling (Soltis and Soltis 2009). As a result, allopolyploids may possess genetic novelties stemming from hybridization. Moreover, the results of allopolyploidization events are often greater than the sum of hybridization and genome doubling, causing complex genomic interactions linked to the fate of new hybrid individual(s) as well as to any evolutionary lineages that result (Qiu et al. 2020). It has been suggested that hybridization may facilitate adaptation to WGD (Marburger et al. 2019), which, in turn, allows hybrids to thrive by restoring fertility (Charron et al. 2019) and fixing heterosis (Qin et al. 2021). Numerous studies have shown that allopolyploidization results in greater genetic variability than autopolyploidization, due to resulting extensive genetic changes or genome reshuffling (reviewed in Feliner et al. 2020). Therefore, although both allopolyploidization and autopolyploidization have likely been prevalent during the evolutionary history of angiosperms (Soltis et al. 2007; Barker et al. 2016), allopolyploidy should, theoretically, have facilitated greater raw material for evolutionary adaptation. However, whether this evolutionary influence of allopolyploidization occurs in nature remains an open question (Soltis et al. 2010).

Due to its outstanding range of morphological diversity and ecological specialization, Gesneriaceae represents an ideal model to understand the nature of the processes underpinning evolutionary radiations, including WGD and hybridization. Gesneriaceae comprises c. 150 genera and 3700+ species in the order Lamiales (Weber et al. 2020). The family is widely distributed across the tropics and subtropics, although most individual gesneriad species have relatively narrow distributions. Many are saxicolous, particularly on limestone, with others epiphytic or terrestrial (Supplementary Fig. S1a–d). Within Gesneriaceae, habits range from rosettiform herbs to lianas, shrubs, and small trees (Supplementary Fig. S1a–d). The flowers are highly specialized, colorful and showy, reﬂecting adaptation to a wide range of pollinators including birds, bats, and bees (Supplementary Fig. S1e–h; Gao et al. 2006; Martén-Rodríguez et al. 2009). Many gesneriads are also commercially important ornamentals, including the African violets (Supplementary Fig. S1i; *Streptocarpus* spp.), gloxinia (Supplementary Fig. S1j; *Sinningia* spp.), and some species of *Primulina* (Supplementary Fig. S1k). Previous studies of lineage diversification in Gesneriaceae have focused primarily on ecological factors, such as palaeoenvironment (Kong et al. 2017, 2022), geographic isolation (Perret et al. 2007; Clark et al. 2009), and pollination syndrome (Roalson and Roberts 2016; Serrano-Serrano et al. 2017), as possible driving forces. However, the underlying genetic mechanisms of evolutionary radiation in the gesneriads have not, to our knowledge, been studied to date.

The Gesneriaceae has likely experienced a complicated genomic evolution based on its wide range of chromosome numbers, from 2*n* = 8 to 2*n* = 144 (Möller and Kiehn 2003; Siljak-Yakovlev et al. 2008). However, little is known about genome duplication dynamics and its evolutionary consequences in the family. Recently, using a high-quality genome of *Primulina huaijiensis* and eight transcriptomes of other gesneriads, Feng et al. (2020) detected a lineage-specific WGD (referred to as the “*D* event”) in subtribe Didymocarpinae, the largest subtribe in Gesneriaceae with about 57% of species. More recently, Nishii et al. (2022), comparing the genome of *Streptocarpus rexii* with *P. huaijiensis,* suggested that the *D* event might be shared with subtribe Streptocarpinae. However, due to limited taxon sampling, the exact phylogenetic placement of the *D* event is still uncertain. Here, we use dense taxon sampling to investigate the nature of this polyploidization event and to indicate whether it contributed to the evolutionary success of this particular gesneriad lineage.

Testing hypotheses regarding the long-term effects of polyploidy requires a robust phylogenetic framework. Numerous molecular phylogenetic studies of Gesneriaceae over the last two decades, primarily focused on circumscriptions and relationships among subgroups in the family, have yielded dramatic generic redefinitions (reviewed in Möller and Clark 2013). Weber et al. (2013) proposed a new classification of Gesneriaceae into 3 subfamilies, 7 tribes, and 24 subtribes based on phylogenetic studies. The relationships among the three subfamilies are clear, with the monotypic Andean genus *Sanango* comprising one subfamily (Sanangoideae), sister to the rest of the family, with subfamilies Gesnerioideae (a predominantly New World lineage) and Didymocarpoideae (a predominantly Old World lineage) sister to each other. However, the major phylogenetic relationships below the subfamily level are still unresolved, possibly due to limited phylogenetic signals from the use of a small set of chloroplast and/or nuclear genes (Möller and Clark 2013; Roalson and Roberts 2016). In particular, relationships between subtribes in the tribe Trichosporeae are uncertain (Möller and Clark 2013; Weber et al. 2013).

Genomic-scale data provide an exceptional opportunity for resolving contentious relationships across the tree of life. For instance, transcriptome sequencing, also known as RNA sequencing (RNA-seq), is among the most cost-effective approaches for generating thousands of homologous loci for a large number of individuals and has proven to be a robust tool for phylogenetic analyses at both recent and deep timescales (see Cheon et al. 2020; for an entry into this literature). In addition to phylogenetic analyses, transcriptome data are suitable for identifying ancient WGD events given sound homology inferences under dense taxon sampling (Li et al. 2015; Huang et al. 2016; Yang et al. 2018). This has been demonstrated with large data sets, for instance via the One Thousand Plant Transcriptomes project, through which hundreds of WGD events have been identified in land plants (One Thousand Plant Transcriptomes Initiative 2019). Although these large data sets (in terms of loci), often exhibit high levels of gene tree-species tree discordance, they may allow for a better understanding of evolutionary history during species diversification because the discordance often arises from biological (as opposed to methodological) processes, such as gene duplication and loss, incomplete lineage sorting (ILS) and gene ﬂow (Jeffroy et al. 2006; Degnan and Rosenberg 2009). Moreover, recent developments in bioinformatics allow for more precise identification of the timing and location of WGDs within the tree of life (Thomas et al. 2017; Li et al. 2018; Zwaenepoel and Van de Peer, 2019).

In the present study, we generated new genomic resources and analyzed 138 transcriptomes and four genomes to identify WGD events in the history of Gesneriaceae and investigate their macroevolutionary impacts on diversification. Specifically, we aimed to: 1) reconstruct a robust backbone phylogeny of Gesneriaceae at the subtribal level, 2) investigate the extent and causes of discordance among gene trees, including between plastid and nuclear genomes, 3) infer genome duplications along the backbone phylogeny of Gesneriaceae and study the nature of polyploidy events, and 4) evaluate whether any WGDs detected were associated with diversification rate shifts. Our analyses shed new light on the phylogenetic history of Gesneriaceae and provide evidence that an ancient allopolyploidization event occurring around the Oligocene–Miocene boundary may have facilitated the explosive diversiﬁcation of core Didymocarpinae (i.e., excluding *Henckelia*, *Microchirita*, and *Condonoboea*; *sensu* current study).

## Materials and Methods

### Taxon Sampling, Transcriptome Sequencing and Assembly

We sampled 138 individuals from 137 species of Gesneriaceae, representing 19 of the 24 subtribes (Supplementary Table S1). The monotypic subfamily Sanangoideae and two tribes (Napeantheae and Beslerieae) in the subfamily Gesnerioideae were not included in this study. In addition, we included seven species from Calceolariaceae, Lamiaceae, Plantaginaceae, Bignoniaceae, and Oleaceae as outgroups, based on the phylogeny of Lamiidae estimated by Refulio-Rodriguez and Olmstead (2014). Thus, in total 145 species were sampled, of which 124 comprised newly sequenced transcriptomes, with 7 genomic and 14 transcriptomic sequences obtained from NCBI or other databases (Supplementary Table S1). RNA extraction, sequencing, assembly, and translation followed our previous protocols (Feng et al. 2020; Kong et al. 2022; see Supplementary Methods for details). New sequence reads are deposited in the NCBI Sequence Read Archive (SRA); accession numbers can be found in Supplementary Table S1.

### Orthogroup Circumscription, Orthology Inference and Gene Selection

We circumscribed orthogroups of proteins from all gesneriads and outgroup taxa using the DIAMOND sequence search implemented in OrthoFinder v.2.2.7 (Emms and Kelly 2015). We initially selected orthogroups to be retained for downstream analyses according the following criteria: 1) at least one sequence available per species; 2) mean copy number of the orthogroup ≤ 5; and 3) median copy number of the orthogroup ≤ 2. The 1285 candidate orthogroups were further analyzed using the pipeline of Yang and Smith (2014) to obtain 1:1 orthologs. For each selected orthogroup, we generated alignments in MAFFT v.7.427 (Katoh and Standley 2013) under default parameters and performed a two-round maximum-likelihood (ML) phylogenetic reconstruction in RAxML v.8.2.9 (Stamatakis 2014) under the GTR + Г model. From the resulting gene phylogenies, we pruned terminal branches longer than 0.2 and 10× longer than that (or those) representing its sister clade. We also pruned branches >0.5. Orthology was then inferred based on these homolog trees using the Rooted Tree (RT) method (Yang and Smith 2014). Additional filters were applied to investigate potential sources of errors in gene tree estimation, such as intragenic recombination, hidden paralogs, and long-branch attraction (see Supplementary Methods and Supplementary Fig. S2 for details).

### Low-Copy Nuclear Phylogenetic Analysis

We used both concatenated and coalescent-based methods to reconstruct the phylogeny of Gesneriaceae using resulting alternative data sets. We aligned gene sequences in MAFFT v.7.427 using default parameters and processed each alignment with TrimAL v.1.4.15 (Capella-­Gutiérrez et al. 2009) using the gappyout option to remove poorly aligned regions. To perform concatenated inference, we used ML estimation in IQ-TREE v.1.6.8 (Nguyen et al. 2015), which was set to determine the best-fit partitioning scheme as well as the best-fitting models for each partition using the implementation of ModelFinder (Kalyaanamoorthy et al. 2017). Support was inferred using 1000 Ultra-Fast bootstraps (UFBoot) replicates. We also estimated ML gene trees and 200 bootstraps (BS) topologies from unpartitioned alignments for each locus in IQ-TREE v.1.6.8 using the best-fitting models selected by ModelFinder. For coalescent-based estimations of the species tree, we applied SVDquartets (Chifman and Kubatko 2014) and ASTRAL-III v.5.6.3 (Zhang et al. 2018). For SVDquartets, we used the implementation in PAUP v. 4.0a167 (Swofford 2002) and concatenated matrices to evaluate, at most, 5,000,000 quartets, calculating node support using 200 BS replicates. In ASTRAL-III, we used the best ML gene trees to infer the species tree topology, and 200 gene-tree BS replicates to estimate multilocus BS support (i.e., the parameter “–i –b –r”). Additionally, we estimated a species tree using ASTRAL-Pro (Zhang et al. 2020a) based on 1318 homolog trees generated with the approach of Yang and Smith (2014). ASTRAL-Pro uses paralogs directly to estimate a species tree that is statistically consistent with the multispecies coalescent (MSC) and a birth–death gene duplication and loss model. In ASTRAL-Pro, we used local posterior probability (LPP) to assess clade support.

### Chloroplast Phylogenetic Analysis

We extracted plastid sequences from our transcriptomic assemblies following the pipeline of Gitzendanner et al. (2018); details provided in Supplementary Methods. For each gene matrix obtained, we removed taxa with sequences shorter than 300 bp; we discarded entire matrices if they contained more than 30% missing taxa. This yielded 27 gene sequence matrices, for which we performed the same procedures of alignment, trimming and ML inference as for the nuclear genes. In addition, we applied a Bayesian Inference (BI) approach to estimate the plastid phylogeny, using MrBayes v3.2.7a (Ronquist et al. 2012) running on the CIPRES Science Gateway 3.3 high-performance computing infrastructure (www.phylo.org; Miller et al. 2015). For BI, we determined the best-ﬁt model of evolution for each gene (Supplementary Table S2) using the Akaike information criterion implemented in MrModeltest 3.7 (Posada and Crandall 1998). BI was conducted via two Markov Chain Monte Carlo runs with one cold chain and three heated chains for 10,000,000 generations. Trees were sampled every 1000 generations and the ﬁrst 25% of generations were discarded as burn-in. Posterior probabilities (PP) were taken from 50% majority rule consensus trees generated in MrBayes.

### Detecting Nuclear Gene Tree Discordance

To explore the degree of conflict at each node of a species tree given individual gene trees, we calculated the extended quadripartition internode certainty (EQP-IC) using the program QuartetScores (Zhou et al. 2020). EQP-IC is measured using quartet-based frequencies of alternative quadripartition topologies around a given internal branch in the species tree from a set of gene trees, and has been shown to be more robust than bipartition-based measures (Zhou et al. 2020). We performed analyses in QuartetScores based on data set_2, the data set with the highest signal-to-noise ratio (see “Results” section).

### Detecting the Source of Cytonuclear and Genealogical Conflicts

To investigate if the observed conflict at deep nodes between the plastid and nuclear phylogenies was best explained by hybridization or ILS, we performed a coalescent simulation. To accomplish this, we first assembled a 20-taxon data set, representing almost every subtribe, and estimated nuclear and plastid phylogenies separately in ASTRAL-III and IQ-TREE, respectively (details of sampling strategy and phylogenetic analysis can be found in the Supplementary Methods). Then, following Folk et al. (2017), we simulated 10,000 plastome trees under the MSC model with DendroPy v.4.4.0 (Sukumaran and Holder 2010) using the 20-taxon ASTRAL-III nuclear tree as a model. All branch lengths of the nuclear coalescent tree were scaled by 4.0 to account for expected rates of organellar inheritance (Folk et al. 2017; Morales-Briones et al. 2018). Clade frequencies among the simulated trees were obtained by summarizing them on the observed plastome tree using RAxML v.8.2.9. If ILS alone was responsible for discordance, relationships shown in the empirical plastid tree should be present in the simulated plastid trees with high frequency. By contrast, if hybridization mainly accounts for cytonuclear discordance, we expect that the unique clades of the empirical plastid tree should be absent or at low frequency in the simulated trees.

Recently, Allman et al. (2022) developed a new approach to address gene tree discordance through quartet summaries of gene tree collections. This approach (Allman et al. 2022) involves obtaining a quartet count concordance factor (qcCF) and computing *P*-values to determine the fit of the gene trees to a species tree. We calculated qcCFs for all 365 single-copy gene trees using the function quartetTreeTestInd in the R package MSCquartets (Rhodes et al. 2021). Thereafter, we used the qcCFs to draw simplex plots in MSCquartets under both a model in which the species tree is assumed to be known (model T1) and one in which there is no species tree hypothesis (model T3). We applied the 20-taxon species tree from ASTRAL-III for the T1 model. In these plots, if they reject the MSC model the qcCFs would be plotted far from the model lines (Allman et al. 2022). However, such rejection of the MSC model might be due to either substantial gene tree estimation error (GTEE) or more complicated biological processes, such as gene flow. Therefore, we mapped these rejected quartets back to the ASTRAL-III tree. We expected that if these rejected quartets are a result of gene flow, they should be clustered at specific nodes. In contrast, if the rejected quartets result from GTEE, they should be distributed randomly on the species tree.

### Species Network Analysis

To test the hypothesis that reticulate evolution contributes to observed conflicts, we applied the maximum pseudo-likelihood method (Yu and Nakhleh 2015), implemented in PhyloNet v.3.8.0 (Than et al. 2008), to generate a phylogenetic network based on the 365 single-copy gene trees representing the 20-taxon data set. We performed network searches allowing for 0–4 reticulations and using only nodes in the rooted ML gene trees that had UFBoot support of at least 70%. To determine the best network, we ranked them by log-pseudo-likelihood scores, because pseudo-likelihoods cannot be used in a model ﬁtting framework (Yu and Nakhleh 2015). Due to the difficulties of determining the best network under the pseudo-likelihood-based method in PhyloNet, we also generated a complementary level-1 network using the Network inference Algorithm via NeighbourNet Using the Quartet distance (NANUQ) method (Allman et al. 2019). We determined empirical quartet counts from the gene trees and the network quartet distance between taxa via the NANUQ function in the R package MSCquartets under the parameters “α = 0.01” and “β = 0.95.” Thereafter, we used the NeighborNet algorithms implemented in SplitsTree4 v.4.15.1 (Huson and Bryant 2006) to construct a split graph for visualization based on the quartet distance between taxa.

### D-Statistic Tests for Ancient Introgression

Our network analysis indicated complicated reticulate subtribal relationships in the core Trichosporeae (i.e., Didissandrinae + Streptocarpinae + Loxocarpinae + Didymocarpinae). Therefore, we conducted a *D*-statistic test (also known as ABBA–BABA, Durand et al. 2011) to investigate ancient introgression in this group. To account for uncertainty in relationships within Trichosporeae, we performed analyses using both ASTRAL-III and SVD-Quartets topologies estimated based on data set_2 as guide trees. Based on these two guide phylogenies, we defined all possible four-taxon (subtribal-level) trees in the form of (((P1, P2), P3), O), where O represents an outgroup taxon and P1 and P2 are taxa that are tested for signatures of introgression with P3. We determined the *D*-statistic using the R package evobiR (https://github.com/coleoguy/evobir) based on the concatenated 602-gene alignment. We assembled all possible permutations of species belonging to each of the four test groups, following the guide trees outlined above. For each combination of tests, we performed 200 BS replicates to estimate the standard deviation in *D*-statistic scores and two-tailed *P*-values. We used α = 0.05 as the cutoff for signiﬁcance after applying a Holm–Bonferroni correction for multiple comparisons.

### Inferring Genome Duplications and the Nature of Polyploidy

We applied a combination of approaches to infer WGD events across the backbone phylogeny of Gesneriaceae, including synonymous substitution rate (*Ks*) estimation, gene tree mapping, and syntenic analysis. These analyses used 138 transcriptomes, as well as four Gesneriaceae genomes, that is, *P. huaijiensis* (Feng et al. 2020), *Dorcoceras hygrometricum* (≡ *Boea hygrometrica*; Xiao et al. 2015; Puglisi et al. 2016), *S. rexii* (Nishii et al. 2022), and *Henckelia pumila*.

The genome of *H. pumila* is newly sequenced in this study. We generated 227.71 Gb PacBio long reads, 102.27 Gb Illumina short reads, and 170.42 Gb Hi-C sequencing reads. Using these sequences, we followed our previous protocols to perform genome assembly, assessment, and annotation (Feng et al. 2020, 2021; see Supplementary Methods for details). The genome assembly of *H. pumila* and all sequencing data are deposited in GenBank under BioProject PRJNA882983.

For *Ks* analysis, we used the WGD package (Zwaenepoel and Van de Peer 2019). We constructed *Ks* distributions (ranging from 0.05 to 3) among paralogs from all transcriptomes and four genomes using the default parameters in WGD. For the four Gesneriaceae genomes, we pruned paralogs based on a colinearity analysis using i-ADHoRe (Proost et al. 2012) according to their annotations. We then fitted a Gaussian mixture model to the *Ks* distribution, with the optimal number of components assessed using the Bayesian information criterion. We further compared the divergence among paralogs (due to putative WGD) to the divergence among orthologs in species pairs. To avoid the influence of varying evolutionary rates among genes, we only compared *Ks* distributions for paralogs and orthologs within the same orthogroup. We isolated the *Ks* distributions of paralogs and orthologs with 95% probability, respectively, and estimated the mean and variance of these *Ks* distributions.

For gene-tree-mapping analysis, we applied three different methods. First, we used the Multi-Axon Paleopolyploidy Search (MAPS) algorithm (Li et al. 2018) to map gene duplication onto the phylogeny. We designed a total of four MAPS analyses, one for each node on the backbone phylogeny ﬂagged in the *Ks* analysis. Following Li et al. (2018), we generated positive and null models for comparison based on 100 replicates of 1000 simulated gene trees both with and without WGDs. Second, we used the pipeline developed by Yang et al. (2018) to investigate gene duplication within the tribe Trichosporeae. Unlike MAPS, Yang et al. (2018) method requires no stepwise branching phylogeny. Therefore, we used all sampled taxa in the tribe Trichosporeae (with five species from the core Didymocarpinae), giving a total of 44 taxa. Lastly, to further test for the nature (allopolyploidy or autopolyploidy) of polyploidization at focal nodes highlighted by previous analyses, we used GRAMPA [Gene-tree Reconciliation Algorithm with Multilabeled (MUL) trees for Polyploid Analysis, Thomas et al. 2017] v.1.3. We performed four GRAMPA analyses, each corresponding to the relevant MAPS analysis. We also ran GRAMPA using the 44-taxon data set to further verify the allopolyploid origin of core Didymocarpinae. Details of these gene tree mapping analyses are provided in the Supplementary Methods.

For syntenic analysis, we used MCScan (Python version; https://github.com/tanghaibao/jcvi/wiki/) to compare genomic synteny among the four Gesneriaceae genomes using their assembly and annotation as the input files.

### Divergence Time Estimation

At present, Bayesian methods of divergence time estimation remain constrained by computation times when using genome-wide data. Therefore, we applied the non-Bayesian method, RelTime (Tamura et al. 2012), implemented in MEGA-X (Kumar et al. 2018) to perform molecular dating based on the concatenated 602-gene alignment. For this analysis, we used the GTR + Γ model of nucleotide substitution, assumed the “local clocks” model, set the branch swap ﬁlter to “None” and constrained the topology to the ASTRAL-III tree estimated based on data set_2. Within RelTime, we used minimum and maximum calibration bounds without an underlying probability distribution. Due to a lack of known fossils for Gesneriaceae, we applied secondary calibrations on node age from Roalson and Roberts (2016). Five calibration points were used, distributed throughout the tree: three deep nodes [the crown age of core Gesneriaceae (48.2–77.06 Ma) (*sensu*Roalson and Roberts 2016), the crown age of Gesnerioideae (40.3–66.2 Ma) and the crown age of Didymocarpoideae (46.61–75.03 Ma)] and two relatively shallow nodes [the crown age of Columneinae (20.29–25.70 Ma) and the crown age of Didymocarpinae (21.96–51 Ma)].

### Diversification Rate Shift

We investigated diversification rates and rate shifts using Modeling Evolutionary Diversification Using Stepwise Akaike Information Criterion (MEDUSA, Alfaro et al. 2009) as implemented in the R package geiger v.2.0 (Pennell et al. 2014). To compensate for our incomplete and unbalanced sampling of Gesneriaceae, we pruned the dated tree to a representative species of each subtribe (including two basal lineages within the subtribe Didymocarpinae, that is, the *Henckelia*_*Microchirita* clade and *Condonoboea*). We obtained species richness data for each terminal clade of this pruned tree from Weber et al. (2020). Based on this pruned tree and species richness information, we performed the MEDUSA analysis under a mixed model allowing the program to select between pure birth and birth–death for each shift according to AIC scores corrected for sample size.

## Results

### Nuclear Assembly and Phylogenetic Analyses

To obtain nuclear gene sequences for phylogenetic reconstruction and WGD detection, we sequenced 124 new transcriptomes of Gesneriaceae. Summary RNA-seq statistics are shown in Supplementary Table S1. Initially, we obtained 898, 1:1 putative orthologs representing at least 72 taxa each. Applying the RT method resulted in the loss of two outgroup taxa, *Fraxinus excelsior* and *Olea europaea*, which reduced our sample to 143 accessions. Incorporating additional criteria to accommodate different potential systematic biases (see Supplementary Methods) resulted in four smaller data sets of 602, 353, 289, and 185 loci, respectively (Supplementary Fig. S2). The characters of all five nuclear data sets are provided in Supplementary Table S3, and the extent of missing data for each gene and taxon is presented in Supplementary Table S4.

**Figure 1.**
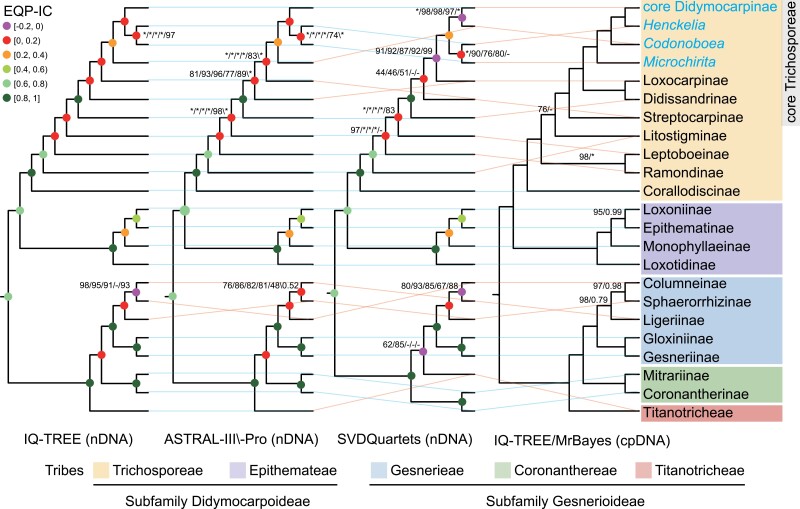
Summary of topological differences involving the major lineages of Gesneriaceae among different methods (i.e., IQ-TREE, ASTRAL-III, ASTRAL-Pro, SVDquartets, and MrBayes) and data sets (i.e., data set_1–6 and the 1318 homologs data set; details of these data sets are provided in Supplementary Table S3). Numbers above internal branches show support values from the different methods respectively [i.e., UFBoot/IQ-TREE, BS/ASTRAL-III, local posterior probability (LPP)/ASTRAL-Pro, BS/SVDquartets, posterior probability (PP)/MrBayes]; asterisks indicate full support. Dashes indicate support values cannot be given due to topological discordance between methods or data sets. All unlabeled internal branches received full support. Topologies 1–3 (from left to right) were estimated based on nDNA (data sets_1–5) using various methods. Support values for topologies 1 and 3 are presented in the order data set_1/ data set_2/ data set_3/ data set_4/ data set_5, whereas the support values for topology 2 were presented in the order data set_1/ data set_2/ data set_3/ data set_4/ data set_5/1318_homologs_data set. Topology 4 is estimated based on cpDNA using IQTREE and MrBayes, with support values presented in the order IQTREE/MrBayes. Colored dots on nodes indicate the extended quadripartition internode certainty (EQP-IC) score. Taxa belonging to the subtribe Didymocarpinae are highlighted in blue.

Our phylogenetic analyses using multiple data sets and methods of inference resolved all tribes, subtribes, and most genera represented by two or more samples, as monophyletic groups with full or nearly full support (Fig. 1 and Supplementary Figs. S3–S8), with the exception that two species in subtribe Ramondinae did not form a monophyletic group in the phylogeny estimated by SVDQuatests based on data set_5 (Supplementary Fig. S7c).

However, the topologies of these phylogenies showed different degrees of conflict depending on the input data set and method of inference (Supplementary Fig. S9). Phylogenies estimated from data set_2 always possessed the highest average support and lowest average RF value compared with other data sets, indicating that this data set has the highest signal-to-noise ratio.

Although the data sets exhibit some conflict and low support (BS, UFBoot or LPP < 70) at shallow nodes (especially within core Didymocarpinae), the backbone topologies of all phylogenies were largely congruent with one another. In particular, the concatenated phylogenies (IQTREE) and the MSC-based phylogenies (ASTRAL-III and ASTRAL-Pro) recovered the same backbone topology of Gesneriaceae with high support, except for the position of Sphaerorrhizinae (Fig. 1). In contrast, the SVDQuartets method always yielded considerably different subtribal relationships within core Trichosporeae and incongruence with other methods around the position of Titanotricheae (Fig. 1).

### Chloroplast Assembly and Phylogenetic Analyses

To reconstruct the plastid phylogeny of Gesneriaceae, we recovered a total of 27 protein-coding chloroplast genes (Supplementary Table S2) from our transcriptomic assemblies. The concatenated chloroplast alignment contained approximately 14% missing data and comprised 21,901 sites, including 4201 (19%) parsimony-informative sites.

As with the nuclear phylogenetic results, the chloroplast phylogeny resulting from both ML and BI methods resolved all tribes, subtribes, and most genera represented by two or more samples, as monophyletic groups with full or nearly full support (Fig. 1 and Supplementary Fig. S10). The topologies recovered by ML and BI were relatively congruent (RF = 17) but contained many poorly supported nodes (UFBoot < 70 or PP < 0.7], primarily within Gloxiniinae and core Didymocarpinae. The backbone topology of the cpDNA phylogenies shows several conflicting relationships compared with the nuclear backbone topologies, especially concerning relationships among major lineages within core Trichosporeae (Fig. 1). Additionally, the highly supported sister relationship between Ramondinae and Leptoboeinae in the cpDNA phylogenies was never recovered in nuclear species trees, whereas the placement of Litostigminae in the cpDNA phylogenies is ambiguous: the ML cpDNA phylogeny revealed that Litostigminae was sister to the core Trichosporeae, but with low UFBoot (70%); the 50% majority rule consensus of the BI results shows Litostigminae, Leptoboeinae + Ramondinae and core Titanotricheae forming a polytomy.

### Substantial Gene Tree Discordance

In our data set, results from the QuartetScores analysis revealed extensive gene tree discordance. Regardless of which topology from among the three nuclear species trees was used as a reference, the QuartetScores pipeline found that ca. 39% of nodes in the reference tree possessed EQP-IC values between –0.2 and 0.2 (Supplementary Fig. S11). This result indicates that a large proportion of the sampled quartets for these nodes were best suited to alternative topologies compared with the reference. Notably, most nodes along the backbone phylogeny of tribe Trichosporeae had especially low EQP-IC scores (–0.2 < EQP-IC < 0.2) (Fig. 1). However, nodes at the base of all tribes, subtribes, and most genera had relatively high EQP-IC values (>0.5, Fig. 1 and Supplementary Fig. S4), suggesting somewhat concordant signals among genes at these taxonomic levels.

### ILS Alone Cannot Explain Either Cytonuclear or Genealogical Discordance

**Figure 2.**
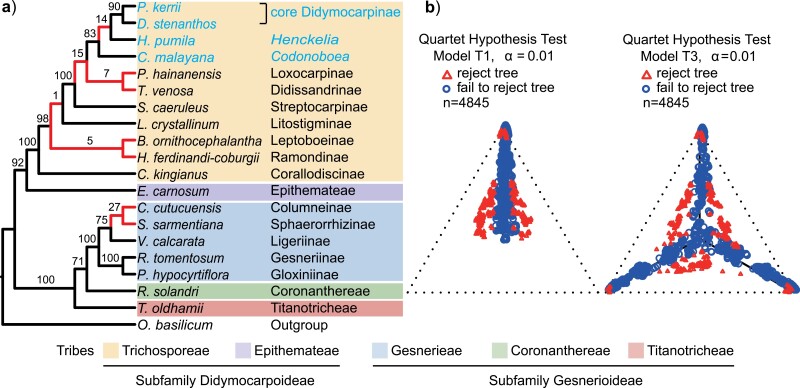
Detecting the source(s) of cytonuclear and genealogical discordance based on a reduced, 20-taxon data set. a) Summary of results of plastome tree simulations. Red internal branches indicate clades unique to the chloroplast topology compared with the nuclear species tree. Numbers above internal branches show clade frequency (%) among simulated plastid genes. b) Simplex plots of quartet count concordance factors (qcCFs) for the 365 gene trees. Species full names: *P. kerrii*, *D. stenanthos*, *H. pumila*, *Codonoboea malayana*, *Paraboea hainanensis*, *Tribounia venosa*, *S. caeruleus*, *L. crystallinum*, *B. ornithocephalantha*, *H. ferdinandi-coburgii*, *Corallodiscus kingianus*, *Epithema carnosum*, *C. cutucuensis*, *S. sarmentiana*, *Vanhouttea calcarata*, *Rhytidophyllum tomentosum*, *Pearcea hypocyrtiflora*, *Rhabdothamnus solandri*, *Titanotrichum oldhamii* and *Ocimum basilicum*. Taxa belonging to the subtribe Didymocarpinae are highlighted in blue.

To dissect the cause of the observed conflicts among gene trees and between nuclear and chloroplast trees, we performed several analyses using a reduced 20-taxon data set representing all major lineages in Gesneriaceae (see Supplementary Fig. S4b). The 20-taxon ASTRAL-III tree (Supplementary Fig. S12) based on 365 single-copy nuclear genes recovered a topology largely consistent with the ASTRAL-III tree using all taxa (Supplementary Fig. S4b) except for two conflicting relationships. The 20-taxon IQ-TREE tree (Fig. 2a) based on 27 chloroplast genes recovered a topology fully consistent with the ML tree based on all taxa (Supplementary Fig. S10). The coalescent simulation found that all unique clades in the empirical plastid tree had low frequency (no more than 15%) among simulated plastome trees, with the exception that 27% of simulated plastome trees agreed with the sister relationship between *Corytoplectus cutucuensis* and *Sphaerorrhiza sarmentiana* (Fig. 2a). Thus, this result favors a hybridization scenario to account for plastid and nuclear phylogenetic incongruence. Furthermore, the statistics for qcCFs also suggested that hybridization should be considered as a likely cause for the conflicts among the nuclear gene trees, in addition to ILS. About 8.98% of tests based on both the T1 and T3 models rejected the MSC hypothesis at α = 0.01 level (Fig. 2b). Mapping these outlier quartets back to the species tree revealed that most of them were concentrated at several specific nodes (Supplementary Fig. S12), indicating that gene flow rather than GTEE underlies these rejected qcCFs. Nevertheless, a large number of qcCFs were plotted on or near the T1 or T3 model lines, with some of these near the centroids of the simplexes (Fig. 2b). This indicates that ILS likely also represents a considerable source of gene tree discordance.

### Widespread Ancient Hybridization and Introgression in Gesneriaceae

**Figure 3.**
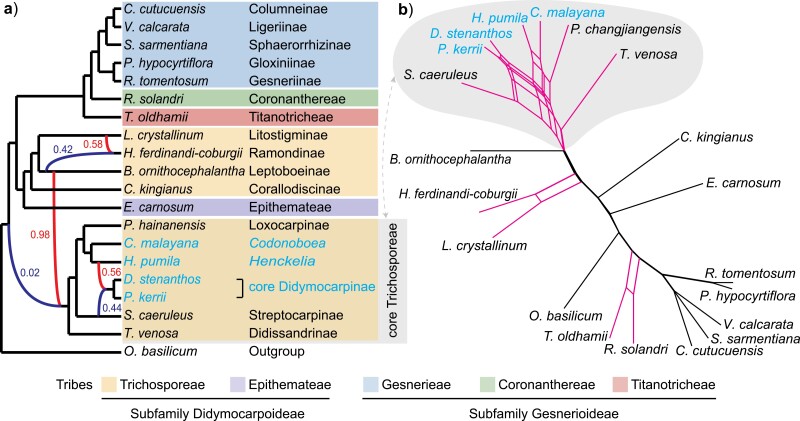
Species networks derived from the reduced, 20-taxon data set. a) Best species network inferred from PhyloNet based on pseudo-likelihood analyses. Red and blue lines indicate greater and lesser parental contributions, respectively, to hybrid lineages. Numbers adjacent to branches indicate inheritance probabilities for each hybrid node. b) Species network inferred by NANUQ. Pink branches indicate areas of possible reticulation. For full species names see legend to Figure 2. Taxa belonging to the subtribe Didymocarpinae are highlighted in blue.

We used wo methods to estimate a phylogenetic network to determine whether reticulate evolution may have contributed to phylogenetic conflicts. The PhyloNet analyses suggested three reticulations in Trichosporeae as the best ﬁt (Fig. 3a); of these, two suggest “classic” hybrid speciation events with inheritance probabilities (γ) from both parental lineages near 0.5. Of these two, *Haberlea ferdinandi-coburgii* arose from hybridization between *Boeica ornithocephalantha* (γ = 0.42) and *Litostigma crystallinum* (γ = 0.58), whereas *Didymocarpus stenanthos* and *Petrocosmea kerrii* were derived from hybridization events between *H. pumila* and *Streptocarpus caeruleus* (γ = 0.56 and 0.44, respectively). The third reticulation event comprised highly biased gene flow between an ancestor of the whole Gesneriaceae family (γ = 0.02) and the core Trichosporeae (γ = 0.98). As expected, the NANUQ method revealed a more reticulate topology, with all species in the core tribe Trichosporeae, in particular, related to a complicated network structure (Fig. 3b).

To further elucidate the reticulate (or unresolved) subtribal relationships in the core Trichosporeae, we tested for ancient introgression among major lineages of this group using the *D*-statistic test. In total, we defined 26 four-taxon subtribal-level trees based on 2 topologies of core Trichosporeae, resulting in a total of 19,691 combinations (for details see Supplementary Table S5). In combination with the PhyloNet and NANUQ results, analysis of the *D*-statistic test suggests a scenario of complex ancient introgression in the core Trichosporeae (Supplementary Fig. S13). Sixteen out of 26 tests showed significant gene flow (more than 50% of combinations had *Z* > 3 and adjusted *P* < 0.05) and only 4 tests showed support for insignificant gene flow (fewer than 10% of combinations had *Z* > 3 and adjusted *P* < 0.05). A summary of all *D*-statistic tests is available in Supplementary Table S6.

### Ancient WGD Events in Gesneriaceae

**Figure 4.**
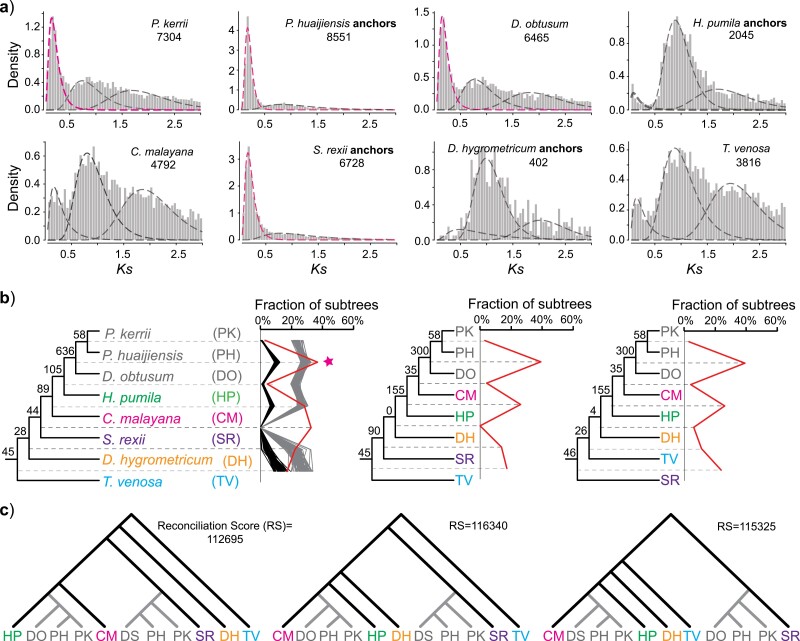
Identiﬁcation, phylogenetic placement, and allopolyploid origin of the *D* event [a lineage-specific whole genome duplication (WGD) shared by core Didymocarpinae]. a) *Ks* distribution for paralogs in eight gesneriads (i.e., *P. kerrii*, *P. huaijiensis*, *Didymostigma obtusum*, *H. pumila*, *C. malayana*, *S. rexii*, *D. hygrometricum*, and *T. venosa*). Gaussian distributions produced by mixture models are shown as dashed lines on each *Ks* plot with inferred Gesneriaceae-specific WGDs highlighted in pink. Numbers under taxon names indicate the total number of paralog pairs used for the *Ks* plot. b) MAPS results from observed data (red line), null (black lines), and positive (gray lines) simulations on the portion of the phylogeny surrounding the *D* event. The three topologies represent the different guide trees used in test 4 of the MAPS analysis. Colors indicate species with differing placements among the three topologies. Numbers above internal branches indicate the number of gene subtrees that support a shared gene duplication at this node. The pink star indicates a putative WGD at the corresponding node. Brown highlighting indicates species of core Didymocarpinae. c) The optimal multilabeled trees inferred by GRAMPA.

In this study, we used the results of *Ks* plots, orthologous divergence, genome synteny, and three different phylogenomic approaches in a total evidence approach to infer and place ancient WGDs within the context of the evolutionary history of Gesneriaceae. In the *Ks* plots, we found that 74 species showed a significant peak of duplication near *Ks* = 0.18 (Fig. 4a; Supplementary Fig. S14; and Table S8). Specifically, all 53 species examined within core Didymocarpinae possess this *Ks* peak near 0.18. This *Ks* peak is clearly taller than the peak near 0.75 and possibly represents WGDs that occurred after the WGD shared by almost all Lamiales [i.e., that referred to as ANMAα by the One Thousand Plant Transcriptomes Initiative (2019) and the *L* event in Feng et al. (2020)].

To determine the phylogenetic placements and nature of the putative WGDs revealed by the *Ks* plots, we applied three different gene-tree mapping approaches. In the four independent MAPS analyses, we found in total 13 nodes that showed statistically significant gene duplications compared with the null model and, of these, 9 were also statistically consistent with a simulated model of WGD (Supplementary Fig. S15). In the placement of the *D* event previously suggested by Feng et al. (2020) based on genomic synteny, MAPS revealed that nearly 40% of gene subtrees support a shared gene duplication at the base of core Didymocarpinae, regardless of which guide tree was used (Fig. 4b), suggesting the *D* event is shared by all core Didymocarpinae. However, the method of Yang et al. (2018) did not detect a strong duplication signal at the base of core Didymocarpinae. Rather, it identified the strongest signal at the base of core Trichosporeae (Supplementary Fig. S16). The method of Yang et al. (2018) also identified additional bursts of gene duplication along the backbone phylogeny of Trichosporeae (Supplementary Fig. S16), most of which are consistent with the corresponding MAPS results. We further used GRAMPA to study the nature of the *D* event and other putative WGDs. Regardless of which of the three guide trees was used, GRAMPA revealed a best-scoring MUL tree in which the core Didymocarpinae clade was of allopolyploid origin between Streptocarpinae and *Henckelia* or *Codonoboea* (Fig. 4c). The three alternative scenarios (shown left to right in Fig. 4c) are supported by 1532, 689, and 302 gene family trees respectively, indicating Streptocarpinae and *Henckelia* are more likely to be the parents of the allopolyploid clade than Streptocarpinae and *Codonoboea*. The allopolyploid origin of core Didymocarpinae is also supported by the 44-taxon data set (Supplementary Fig. S17). However, GRAMPA did not infer the other putative WGDs suggested by the *Ks* plots, MAPS, and the method of Yang et al. (2018), and we observed that the singly labeled species tree had a better reconciliation score than the optimal MUL tree inferred by GRAMPA in these analyses.

**Figure 5.**
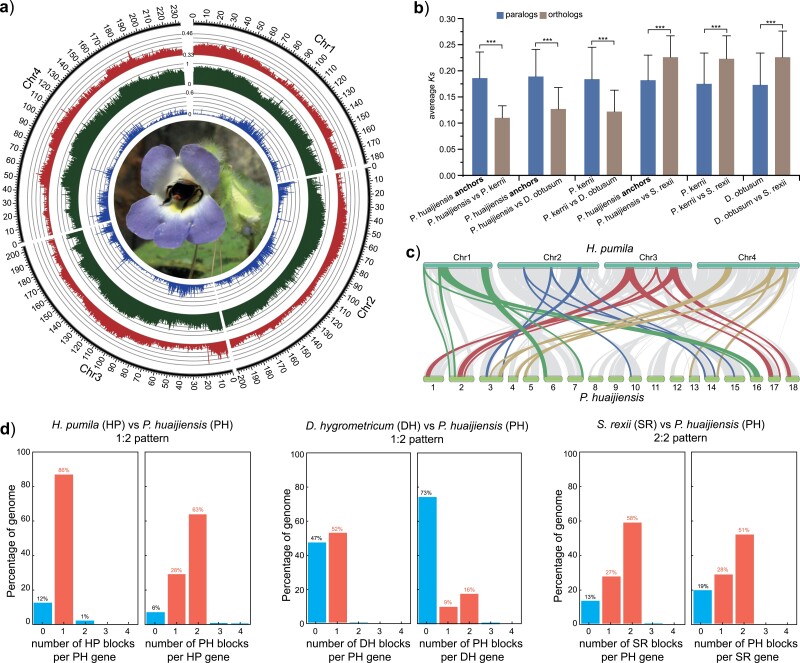
Genome features of *H. pumila* and comparative genomics analyses. a) Genomic features of *H. pumila*. Concentric circles, from outermost to innermost, show guanine-cytosine content (red), transposable element density (green), and gene density (blue). The photo shows a flower of *H. pumila*. b) Comparison of *Ks* distributions between paralogs and orthologs. Data are represented as mean ± SD. Statistical analysis was performed using an unpaired *t*-test. ***P < 0.001. c) Macrosynteny between *H. pumila* and *Primulina huaijienesis* with tracking of genomic positions by color-coded syntenic blocks representing syntenic relationships of 1:2. d) Syntenic depth ratios between *P. huaijiensis* and three other Gesneriaceae genomes (i.e., *H. pumila*, *D. hygrometricum* and *S. rexii*).

To further determine whether the *D* event is specific to core Didymocarpinae or shared within core Trichosporeae, we assembled a chromosome-scale genome of *H. pumila* and carried out comparative genomic analyses. The resulting genome spanned 885.25 Mb, accounting for 98.68% of the estimated genome size based on K-mer analyses (897.04 Mb; Supplementary Fig. S18) with a contig N50 of 5.3 Mb. These contigs were further assigned to four pseudochromosomes by Hi–C analysis (Fig. 5a), consistent with previous chromosome counts of this species (2*n* = 8; Christie et al. 2012). We demonstrated a high consistency and completeness of the assembly using a series of indicators (Supplementary Tables S9–S13), further annotated 65.54% of the genome as repeats (Supplementary Table S14), and identified a total of 27,670 protein-coding genes (Supplementary Tables S15 and S16). Details of the genome assembly, assessment, and annotation are available in the Supplementary Note. Syntenic analysis between *H. pumila* and *P. huaijiensis* revealed a syntenic depth ratio of 1:2, with many large one-to-two syntenic blocks detected (Fig. 5c; Supplementary Fig. S19). However, because the genomes of *D. hygrometricum* and *S. rexii* are highly fragmented, we can only obtain syntenic depth ratios between these two genomes and *P. huaijiensis*, which were 1:2 and 2:2, respectively (Fig. 5d). The syntenic results were consistent with the *Ks* results (Fig. 4a) and revealed that both *P. huaijiensis* and *S. rexii* underwent a recent WGD, whereas both *H. pumila* and *D. hygrometricum* lack a recent WGD after the *L* event. Further comparison within and between-species *Ks* show that the average *Ks* values of paralogs of core Didymocarpinae species are significantly older than the average *Ks* values of orthologs of species pairs within core Didymocarpinae, but significantly younger than the average *Ks* values of orthologs of species pairs between core Didymocarpinae and Streptocarpinae (Fig. 5b). Collectively, our results corroborate the *D* event as specific to the core Didymocarpinae.

**Figure 6.**
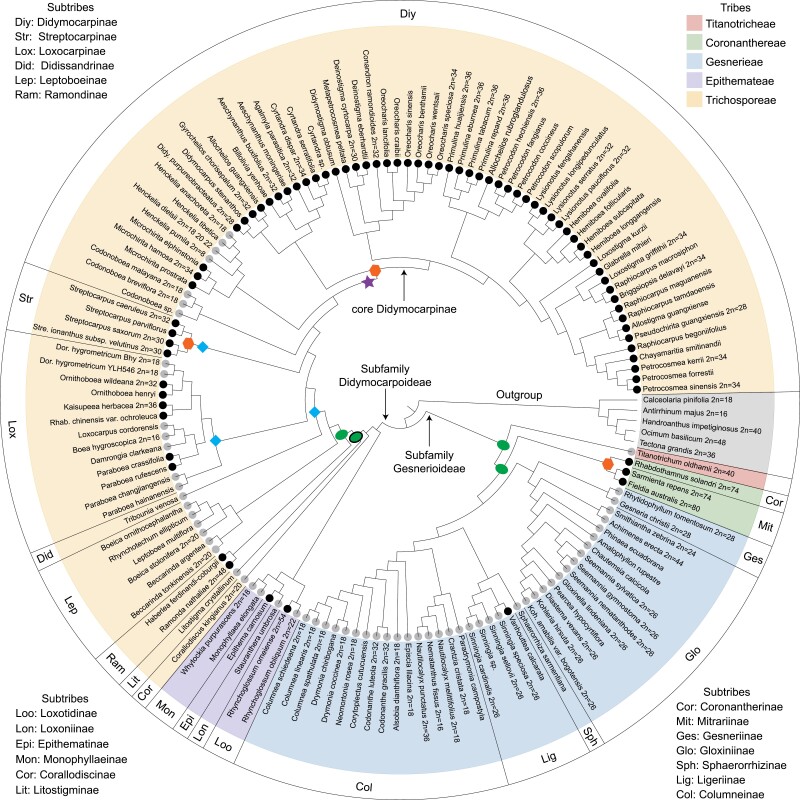
Summary of results for inferring WGDs in Gesneriaceae based on *Ks*, MAPS, the method of Yang et al (2018), GRAMPA, and chromosome number, shown on the ASTRAL-III topology estimated based on the data set_2. Black dots on terminal branches indicate that *Ks* plots of corresponding species possess an obvious WGD peak near 0.18; gray dots indicate corresponding *Ks* plots do not possess the obvious WGD peak near 0.18. Orange polygons represent putative WGDs supported by MAPS, *Ks*, and chromosome numbers. Green ovals indicate significant increases in gene duplications inferred by MAPS analyses. Blue squares indicate significant increases in gene duplications inferred by the method of Yang et al. (2018). Black-edged green ovals indicate significant increases in gene duplications inferred by both MAPS and the method of Yang et al. (2018). A violet star represents the WGD supported by GRAMPA. Chromosome numbers are provided adjacent to the species names and were obtained from WebCyte (Möller and Kiehn 2003; http://elmer.rbge.org.uk/webcyte/webcyteintro.php), CCDB (Chromosome Counts Database; Rice et al. 2015; http://ccdb.tau.ac.il/browse/), and additional literature (Liu et al. 2014; Möller et al. 2016; Zhou et al. 2017; Yang et al. 2020).

The results of WGD identification based on *Ks*, MAPS, the method of Yang et al. (2018), and GRAMPA, as well as chromosome number, are summarized in Fig. 6 and Supplementary Fig. S20.

### Rapid Diversification After Ancient Allopolyploidization in Didymocarpoideae

**Figure 7.**
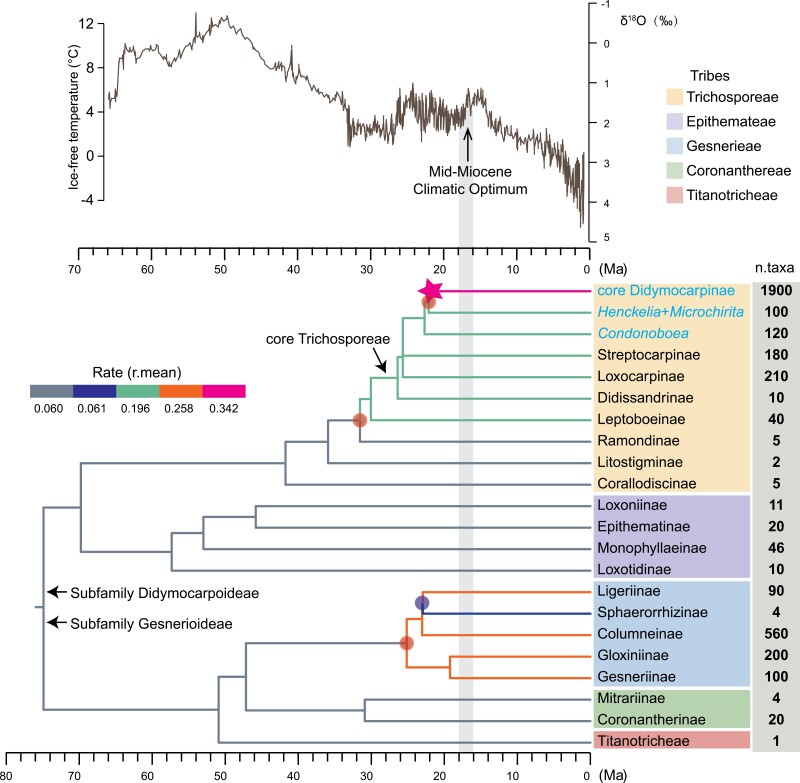
Diversiﬁcation rate shifts in Gesneriaceae detected by MEDUSA. Inferred rate shifts are indicated by red (accelerations) and blue (decelerations) circles. Branch colors indicate the mean diversiﬁcation rate (species/million years; see legend). Figures to the right of taxon names indicate the species richness of each terminal subtribe or clade, based on Weber et al. (2020). The pink star represents the inferred allopolyploidization event. The global climate curve over the last 65 million years (modiﬁed from Zachos et al., 2008) and the geological time scale are shown for reference, with the Mid-Eocene Climatic Optimum arrowed. Taxa belonging to the subtribe Didymocarpinae are highlighted in blue.

The MEDUSA analysis detected four diversification-rate shifts across the backbone phylogeny of Gesneriaceae (Fig. 7): in Gesnerioideae, the diversification rate accelerated at the base of Gesnerieae but slowed significantly along the branch leading to Sphaerorrhizinae. In contrast, Didymocarpoideae showed increasing diversification rates through time, with two lineages marked by significantly accelerated rates: the stem lineage of the Leptoboeinae-core Trichosporeae clade, and the branch leading to core Didymocarpinae. Notably, the diversification rate of the allopolyploid core Didymocarpinae lineage increased to nearly 2.5-fold higher than the background rate (*r* = 0.342 vs. 0.139 species/Ma). Our molecular dating results further showed that the rapid radiation of this allopolyploid lineage occurred during the mid-Miocene Climatic Optimum (MMCO; ~16–18 Ma; Supplementary Fig. S21), with many clades emerging suddenly at this time.

## Discussion

In this study, we demonstrate the utility of transcriptomes for resolving contentious relationships and for the identification of ancient WGD events in a morphologically diverse and ecologically specialized model plant family, Gesneriaceae. Our results are in agreement with previous studies (Cannon et al. 2015; Edger et al. 2015; Li et al. 2015, 2018; Huang et al. 2016; Xiang et al. 2016; Ren et al. 2018; Yang et al. 2018; Cai et al. 2019; One Thousand Plant Transcriptomes Initiative 2019; Zhang et al. 2020b) in underscoring that transcriptomics provides a cost-effective tool for phylogenetic inference and evolutionary analyses. Our results also demonstrate the importance of investigating the nature of WGD (i.e., auto- or allopolyploid) to better understand their evolutionary consequences as well as thoroughly testing for the root sources of conﬂict in phylogenomic analyses. Here, we discuss the level of certainty in, and implications of, the results described above.

### The Allopolyploid Origin of Core Didymocarpinae and Multiple Putative WGDs in Gesneriaceae

An ancient whole genome duplication (the *D* event) was recently inferred using synteny in a chromosome-level genome assembly of *P. huaijiensis* (Feng et al. 2020). Specifically, based on eight species of Gesneriaceae, Feng et al. (2020) placed this event within the subtribe Didymocarpinae. Using transcriptomes and genomes for 80% of genera in Didymocarpinae, our *Ks* plots and orthologous divergence analyses, genomic synteny, and phylogenomic analyses provide strong evidence that the *D* event is shared across core Didymocarpinae. The inference and placement of the *D* event are also supported by chromosomal evidence that all reported chromosome numbers in core Didymocarpinae (2*n* = 28, 30, 32, 34, and 36; Fig. 6 and Supplementary Fig. S20) are close to double the basic chromosome number of the Didymocarpoideae (2*n* = 18, Wang et al. 1998; Möller and Kiehn 2003). We further used GRAMPA to study the nature of this WGD event and found that the core Didymocarpinae represents an allopolyploid clade (Fig. 4c and Supplementary Fig. S17). This finding is congruent with our comparison between phylogenies inferred from cpDNA and nDNA data sets (Fig. 1) and our PhyloNet results (Fig. 3a).

However, the results of within- and between-species *Ks* analyses conflict with those reported by Nishii et al. (2022). Nishii et al. (2022) found that the *Ks* distribution of orthologs between *S. rexii* and *P. huaijiensis* (between-species *Ks*) was similar to the *Ks* distribution of paralogs for these two species (within-species *Ks*), whereas their average between-species *Ks* value was significantly older than the average within-species *Ks* value in our study (Fig. 5b). This might be due to the fact that Nishii et al. (2022) did not consider the different substitution rates among genes when estimating the *Ks* distribution. Although the within-species *Ks* are often compared with between-species *Ks* to infer the relative or absolute timing of WGDs (e.g., Blanc and Wolfe 2004; Li et al. 2018), such comparisons allow meaningful interpretation only if the substitution rates of genes used to derive both distributions are similar (Barker et al. 2008; Shi and Chen 2020). In our study, we compared *Ks* values only between paralogs and orthologs within the same orthogroup, to reduce biases caused by differing substitution rates among genes.

In addition, it is noteworthy that, using the method of Yang et al. (2018), we did not detect a strong duplication signal at the base of core Didymocarpinae, but instead at the base of core Trichosporeae (Supplementary Fig. S16). A shared WGD at the base of core Trichosporeae is not supported by the *Ks* analysis, because many species (i.e., *Henckelia*, *Codonoboea*, Didissandrinae, and several species in Loxocarpinae) in this lineage lack the WGD peak near *Ks* = 0.18 (Fig. S14). In particular, analyzing the high-quality genome of *H. pumila* provides convincing evidence that *H. pumila* lacks a recent WGD after the *L* event. It is unlikely that the absence of the recent WGD signal in *H. pumila* is due to the absolute loss of its paralogs given that the crown age of the core Trichosporeae (assuming a WGD occurred at the base of core Trichosporeae; 26.34 Ma; Supplementary Fig. S20) is similar to the half-life decay period for duplicates in angiosperms (approximately 21.6 Ma; Ren et al. 2018). In addition, the relatively large genome size of *H. pumila* (897.04 Mb) suggests that this genome has not experienced a sharp reduction. One possible way to reconcile the results from the method of Yang et al (2018) and the *Ks* analysis is by invoking the allopolyploid origin of core Didymocarpinae. With an allopolyploidization event, the method of Yang et al (2018) will place the duplication signal at a deeper node than the true position of the allopolyploidization (Yang et al. 2015, 2018), and this is also a major caveat for most current gene-tree mapping methods not accounting for allopolyploidy (Kellogg 2016).

We found multiple bursts of gene duplication across the backbone phylogeny of Gesneriaceae and many recent WGDs at terminal branches based on both *Ks* analyses and other approaches (Fig. 6 and Supplementary Fig. S20). These results appear to confirm previous cytological studies, which have suggested several polyploidization events in Gesneriaceae (Möller and Kiehn 2003). However, these individual polyploidization events were not the focus of our study. Instead, we focused on bursts of gene duplication along the backbone phylogeny of Gesneriaceae, and, by combining *Ks* plots, chromosome counts, and phylogenomic analyses, we found that bursts of gene duplication at the bases of tribe Coronanthereae and subtribe Streptocarpinae may represent WGDs (indicated by orange polygons in Fig. 6 and Supplementary Fig. S20), whereas duplications at other nodes are less likely to be WGDs (indicated by green ovals in Fig. 6 and Supplementary Fig. S20). Future studies using whole-genome synteny should be conducted to confirm the nature of all inferred duplication events. Furthermore, denser taxon sampling will be necessary to fully resolve the phylogenetic placement and origin of these putative WGDs with high precision (Yang et al. 2018).

### Establishment of Ancient Allopolyploidization Coincides with Oligocene–Miocene Boundary Climate Upheaval

WGD events seem not to occur at random through the evolutionary history of angiosperms. Instead, WGDs appear concentrated in geological ages during which strong environmental changes occurred, such as the Cretaceous period (100–120 Ma; Zhang et al. 2020c) and the Cretaceous–Paleogene (K–Pg) boundary (66 Ma; Vanneste et al. 2014; Koenen et al. 2021). Our age estimates suggest that the establishment of the allopolyploid lineage, core Didymocarpinae, occurred around the Oligocene–Miocene boundary (ca. 22 Ma; Supplementary Fig. S21). This coincides well with the previous estimate for the *D* event (20.6–24.2 Ma) based on whole-genome sequence data (Feng et al. 2020). WGDs in other plant families are reported to have occurred around the Oligocene–Miocene boundary, including in Asteraceae (Huang et al. 2016), Cucurbitaceae (Guo et al. 2020), Poaceae (Guo et al. 2019), Fabaceae (Zhao et al. 2021), and others (Ren et al. 2018; Cai et al. 2019; Xia et al. 2022). Together, these results suggest that the Oligocene–Miocene boundary was likely an important geological age associated with WGD events in plants.

Climatic change delivers stressful conditions for plants in particular, due to the sessile lifestyle. Thus, polyploids (especially allopolyploids), which bear genetic novelties, may have an advantage during such times (Van de Peer et al. 2017). The Oligocene–Miocene boundary was characterized by sharp fluctuations in global temperatures and precipitation regimes (Zachos et al. 2008), which may have promoted the dominance of the allopolyploid core Didymocarpinae lineage found in this study. Following the climatic instability of the Oligocene–Miocene boundary, the MMCO represented a period of equitable, tropical, wet climate in eastern, and Southeastern Asia (Sun and Zhang 2008). This may have provided ecological opportunities for the newly derived allopolyploid lineage, thus further facilitating the expansion of core Didymocarpinae.

The relationship between diversification and WGDs has been explored at various taxonomic levels across angiosperms (Tank et al. 2015; Huang et al. 2016; Landis et al. 2018; Ren et al. 2018; Smith et al. 2018; Stull et al. 2021), but drawing a synthesis from existing studies is difficult, in part due to the varying scales of the analyses. Furthermore, without discriminating between the two plausible types of WGDs, conclusions on how WGDs impact the diversification of plants are constrained (Soltis et al. 2010; Madlung 2013; Kellogg 2016). Our study has revealed that the core Didymocarpinae experienced an allopolyploid-type WGD event via hybridization. This allopolyploidization coincided with an increase in diversification rate in the lineage leading to core Didymocarpinae (Fig. 7), which contains about 51% of the species richness of the whole Gesneriaceae family and 76% of the species richness of Didymocarpoideae. Therefore, Gesneriaceae appears to present a case in which ancient allopolyploidization may have facilitated rapid diversification and a dramatic increase in species richness. Similar cases have been found in temperate bamboo (Guo et al. 2019) and legumes (Koenen et al. 2021).

However, it is often difficult to establish that WGD via hybridization caused subsequent diversification (Mitchell and Whitney 2021)—the observed coincidence of allopolyploidization and an upshift in diversification rate can also be explained by other alternative models. For example, both WGD and diversification rate might have been driven by climatic changes, such as at the Oligocene–Miocene boundary, and be otherwise unrelated. Moreover, accurate inferences of diversification processes are still challenging (May and Moore 2016; Moore et al. 2016) where, as in Gesneriaceae, taxon sampling within a medium-large family remains incomplete. More accurate future estimates of diversification shifts might better reveal the relationship between WGDs and diversification.

### Phylotrancriptomics Confirms Relationships in Gesneriaceae and Provides Insights into the Causes of Phylogenetic Conflict

In this study, we obtained hundreds of nuclear genes as well as 27 plastid genes from 138 transcriptomes to reconstruct the phylogeny of Gesneriaceae. Our phylotrancriptomic approach confirmed most tribal and subtribal circumscriptions *sensu*Weber et al. (2013), and the monophyly of many genera in Didymocarpoideae (Möller et al. 2011; Wang et al. 2011; Weber et al. 2011). The backbone phylogenies estimated in our study by both concatenation (IQTREE) and coalescent-based (ASTRAL-III and ASTRAL-pro) methods using different nuclear matrices were largely congruent with recent phylogenomic studies of Gesneriaceae using targeted sequencing (Ogutcen et al. 2021).

Our results supported the monophyly of the subtribe Mitrariinae, which was suggested to be paraphyletic in the phylogeny estimated by Roalson and Roberts (2016) using a few chloroplast and nuclear genes. In our study, the two Mitrariinae genera sampled (*Sarmienta* and *Fieldia*) were recovered as sister groups with full support in all phylogenies (Supplementary Figs. S3–S8 and S10). We also clarified relationships within tribe Epithemateae, with relationships among its four subtribes emerging consistently in all our phylogenies, with full support and high EQP-IC scores (Fig. 1).

However, we identified several nodes across the backbone topologies of both Trichosporeae and Gesnerieae characterized by extensive gene-tree conﬂict (Fig. 1). Interestingly, despite strongly conflicting signals across the backbone topology of Trichosporeae, all our phylogenies recovered the core Trichosporeae clade (including Didissandrinae, Streptocarpinae, Loxocarpinae, and Didymocarpinae) with full support and high EQP-IC values (Fig. 1). We further applied several approaches to dissect the source of the observed discordance. These results provide important insights into the phylogenetic history of Gesneriaceae, which are discussed below.

By including 28 out of the 35 genera of subtribe Didymocarpinae, our study advances our understanding of this largest and least-understood group of Gesneriaceae (Weber et al. 2013, 2020). Both our plastid and nuclear phylogenies consistently recovered four major clades, that is, core Didymocarpinae, *Henckelia*, *Microchirita*, and *Codonoboea*, within Didymocarpinae. However, relationships among these four clades were conflicting between plastid and nuclear phylogenies (Fig. 1), implying a complex, reticulate evolutionary history within Didymocarpinae. The core Didymocarpine as defined in this study is a monophyletic lineage with full support and a high EQP-IC value (Supplementary Figs. S3–S8 and S10). However, phylogenetic relationships within this core lineage are far from fully resolved. Difficulties in resolving relationships within core Didymocarpinae may be due to both WGD resulting from hybridization as well as ILS stemming from rapid radiation. Notably, rapid radiations represent one of the most intractable types of evolutionary phenomena for phylogenetic reconstruction (Whitﬁeld and Lockhart 2007). Indeed, our molecular dating together with diversification-rate analysis showed that this lineage experienced an explosive diversification with many lineages suddenly generated during the MMCO (~16–18 Ma; Fig. 7 and Supplementary Fig. S21). The rapid diversification at the origin of core Didymocarpinae is accompanied by numerous, extremely short, and internal branches which may have provided abundant opportunities for ILS. Future comprehensive studies using comparative genomics to provide a better understanding of genome evolution in the core Didymocarpinae may give valuable insights into how to untangle the backbone relationships of this group.

We detected strong topological conﬂicts between the plastid and nuclear trees, as well as extensive discordance between individual nuclear gene trees and species trees. Several factors can result in gene tree-species tree discordance and conﬂicts between nuclear and plastid phylogenies, including differing methodological and biological processes (Zwickl et al. 2014; Morales-Briones et al. 2018, 2021; Smith et al. 2020). We are less inclined to think that methodological errors yielded the observed conflicts because of our stringent data filtering that improved the signal-to-noise ratio in the data and, thus, likely provided some means for lessening systematic errors (Smith et al. 2020). This is evidenced, in part, by the relatively high UFBoot values (ranging from 70 to 97) for the 602 gene trees, indicating that GTEE might not be a major cause of the gene tree-species tree discordance detected in our study. However, we cannot fully exclude the possibility of misidentification of paralogs as orthologs due to the lack of sequenced true orthologs in transcriptomes (Siu-Ting et al. 2019).

Among the potential biological sources of gene tree-species tree discordance, ILS and hybridization are the most common causes in eukaryotes as often discussed in systematics literature. ILS was once considered the most prominent source of genealogical discordance because it was an unavoidable consequence of neutral population processes (Degnan and Rosenberg 2009). More recently however, hybridization and introgression have both been understood to play major roles in shaping the eukaryotic tree of life, especially among plants (Soltis and Soltis 2009; Whitney et al. 2010; Taylor and Larson 2019). Within Gesneriaceae, both our model-based analysis (Fig. 2b) and the simulation of plastome coalescence (Fig. 2a) provide strong evidence that reticulate evolution is likely an important source of deep genealogical and cytonuclear discordance. In particular, support for this view was provided by mapping the quartets that rejected the MSC model back to the species tree (Supplementary Fig. S12) in the model-based analysis. Moreover, we provide direct evidence that ancient hybridization and introgression are widespread in Gesneriacaeae (Fig. 3 and Supplementary Fig. S13), and these reticulate edges often show strong conflict indicated by low EQP-IC values (Fig. 1). Our inference of the impacts of hybridization and introgression on the evolutionary history of Gesneriaceae is consistent with observations that the family is susceptible to interspecific hybridization under both greenhouse and natural conditions (Arisumi 1964; Morley 1976; Smith et al. 1996; Afkhami-Sarvestani et al. 2012; De Araujo et al. 2021). In fact, Gesneriaceae ranked eighth out of 282 tracheophyte families in terms of propensity to hybridization (Whitney et al. 2010). Consequently, our study highlights the necessity of using integrative approaches to phylogenetic resolution that can accommodate both ILS and hybridization when estimating phylogenetic relationships within Gesneriaceae.

## Conclusions

In this study, we present a comprehensive phylotranscriptomic analysis of Gesneriaceae with dense taxon sampling to elucidate the backbone phylogeny of this plant family. Within our phylotranscriptomic data set, we detected extensive cytonuclear and genealogical discordance, likely due to both ILS and reticulate evolution, based on inferences using cutting-edge, integrative bioinformatics approaches. In particular, we highlight that hybridization and introgression may have played an important role in causing phylogenetic conflicts across the backbone phylogeny of Gesneriaceae. Our study contributes to a growing body of literature emphasizing the importance of differentiating among the root causes of conﬂicting phylogenetic signals in phylogenomic studies (Wang et al. 2019; Smith et al. 2020; Morales-Briones et al. 2021). Furthermore, our study provides strong evidence for the allopolyploid origin of core Didymocarpinae during a period of climatic flux at the Oligocene–Miocene boundary, followed by a rapid diversification during the MMCO. This suggests that ancient allopolyploidization might have set the genetic stage for the evolutionary success of this lineage compared with other gesneriads. Thus our results provided new support for the evolutionary hypothesis linking polyploidy to survival in periods of environmental stress and subsequent taxonomic expansion during ecological optima (Van de Peer et al. 2017). At the same time, rapid radiation (associated with ILS) combined with allopolyploidization (resulting from hybridization) renders core Didymocarpinae recalcitrant to phylogenetic resolution. In future, leveraging gene synteny and chromosome-level genome scaffolds may aid in resolving relationships in this lineage.

## SUPPLEMENTARY MATERIAL

Data available from the Dryad Digital Repository: http://dx.doi.org/10.5061/dryad.98sf7m0kt.
